# Pseudo‐orthostatic tremor in a cat

**DOI:** 10.1111/jvim.17290

**Published:** 2025-04-05

**Authors:** Marende M. de Gier, Dian Schuil, Laurent Garosi, Mark Lowrie, Koen M. Santifort

**Affiliations:** ^1^ Department of Neurology Evidensia Small Animal Hospital Hart van Brabant Waalwijk The Netherlands; ^2^ Dierenkliniek Zelhem Zelhem The Netherlands; ^3^ Vet Oracle Teleradiology Norfolk UK; ^4^ Movement Referrals Preston UK; ^5^ Department of Neurology, Evidensia Small Animal Hospital Arnhem Arnhem The Netherlands

**Keywords:** feline, involuntary movement, movement disorder, postural tremor, slow orthostatic tremor, tremor frequency

## Abstract

An 11‐month‐old male castrated shorthaired cat was presented with a tremor in both pelvic limbs, which only occurred when standing (ie, countering gravity) or during active extension of the stifle. General clinical and neurological examinations were normal aside from the tremor of the pelvic limbs, which disappeared on performing a weight‐bearing lifting test. Needle electromyography of both pelvic limbs in the conscious state confirmed a low‐frequency (6 Hz) tremor. The cat was diagnosed with pseudo‐orthostatic tremor. The tremor amplitude waxed and waned over time. The cat remained ambulatory and playful, but often assumed lateral recumbency and seemed to have difficulty jumping. Treatment with gabapentin (10‐20 mg/kg PO q8h) for 3 weeks followed by clonazepam (0.1 mg/kg PO q12h) for 1 day (with unacceptable adverse effects) did not result in improvement.

**Abbreviations**
EMG: electromyography
OT: orthostatic tremor
WEBLT: weight‐bearing lifting test

## 1 INTRODUCTION

Tremor is defined as an involuntary, rhythmic, oscillatory movement of a body part.^1^ It can be broadly classified as either resting or action‐related, the latter subdivided into postural tremor, isometric tremor, and kinetic tremor.^1^, ^2^ Postural tremor types reported in veterinary medical literature include orthostatic tremor (OT), idiopathic episodic head tremor, and benign idiopathic rapid postural tremor.^2^

Orthostatic tremor is a generalized high‐frequency (13‐18 Hz) isolated tremor syndrome that occurs when standing.^1^ Confirmation of the tremor frequency is needed because the frequency is included in the definition of the term. Typically, confirmation is accomplished using electromyography (EMG). Orthostatic tremor syndromes in humans can be subdivided into primary OT and primary OT plus, the latter associated with various (often neurodegenerative) disorders including Parkinson's disease.^1^ In primary OT and primary OT plus, tremors occur either without or with other neurological signs, respectively.^1^, ^3^ Of note, a study in humans reported a frequency <13 Hz in 50% of the included cases.^4^ Because frequency is part of the definition of OT, other terms are proposed for this tremor syndrome. Pseudo‐orthostatic tremor (pseudo‐OT) is an OT with a low frequency (<13 Hz), that can be accompanied by other neurological signs.^1^, ^3^, ^4^ It too can be associated with underlying neurological disorders.^1^, ^4^

In veterinary medicine, little is known about OT. A number of case reports and a single large retrospective study describe OT in dogs.^5^, ^6^, ^7^, ^8^, ^9^, ^10^, ^11^ The clinical diagnosis of OT in dogs is based on a combination of clinical signs and findings as well as needle or surface EMG in the conscious state while standing. Clinical signs and findings include the presence of the tremor when weight‐bearing and absence when non‐weight‐bearing, presence of a helicopter sign, and absence or decrease of the tremor when performing a weight‐bearing lifting test (WEBLT).^5^ A helicopter sign is defined as a low‐pitched noise that sounds like a helicopter in flight when auscultating the muscles of the tremoring limb using a stethoscope, reflecting high tremor frequency.^2^, ^5^ Electromyography performed while standing is the gold standard for corroborating the diagnosis and characterizing the tremor (ie, determining its frequency) in veterinary reports, whereas in humans accelerometry also is accepted.^2^, ^3^

Although different types of tremors have been reported in cats, no cases of postural tremors or, specifically, OT have been reported in cats.^12^

## 2 CASE DESCRIPTION

An 11‐month‐old intact male domestic shorthaired cat (body weight, 4 kg) was presented to the neurology department at Evidensia Small Animal Hospital Arnhem (Evidensia Dierenziekenhuis Arnhem) for evaluation of chronic progressive tremors since 8 months of age. The owner (a veterinarian and coauthor [DS]) described it as a tremor of both pelvic limbs that only occurred when standing (ie, countering gravity). The tremors were visible when the cat was standing still and disappeared when walking during the protraction phase of each limb, respectively (Video 1). Tremors were not observed when the cat was sleeping or when it was relaxed in a lying or sitting position. The cat did not have difficulty sitting or rising. The cat did not show a “dancing sign” or wide‐based stance as reported in dogs with OT.^5^

**VIDEO 1:** Video medley of the cat diagnosed with pseudo‐orthostatic tremor. The tremors only occur when the cat is standing still or when the respective limb is weight‐bearing during ambulation. The tremors also occur when the cat is placed in lateral recumbency and external force is applied to the plantar surface of the paw. When the stifle is immobilized and the paw is relaxed, the tremors disappear. In a video taken after 6 months, the cat showed a wide‐based stance and had difficulties jumping.

General clinical examination and neurological examination were unremarkable apart from a subjectively low‐frequency postural tremor of the pelvic limbs that occurred when standing. The tremors were noticeable in the limb supporting weight during ambulation. The tremor was absent in each limb during the protraction phase. The tremors disappeared in both limbs when performing a WEBLT and in lateral recumbency when the limb was relaxed and the stifle immobilized (Video 1). A slow helicopter sound could be auscultated. The tremors also occurred when external force was applied to the plantar surface of the paw when the cat was in lateral recumbency.

Differential diagnoses considered included postural tremor (specifically pseudo‐OT), postural (orthostatic) myoclonus (pending confirmation of visually observed rhythmicity for confirmation of tremor), essential tremor (benign idiopathic rapid postural tremor), neuropathic tremor, and restless legs syndrome.

**FIGURE 1:**
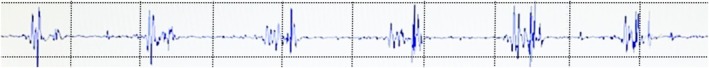
Tracing of needle electromyography in the conscious state while standing of the cat with pseudo‐orthostatic tremors. Electromyography revealed rhythmic muscle discharges of a frequency of 6 Hz in the pelvic limbs (0.3 mV/Division, 100 ms/Division).

A CBC, serum biochemistry, and serum electrolyte concentrations performed by the general practitioner (DS) showed no abnormalities. In the referral hospital, needle EMG of the quadriceps femoris and cranial tibial muscles of the pelvic limbs, while the cat was standing, showed rhythmic muscle discharges at a frequency of 6 Hz (Figure 1 and Video 2). Electromyography of the triceps brachii of the thoracic limbs showed electrical silence; no muscle discharges consistent with tremor were observed. The quadriceps of the left pelvic limb were tested again in lateral recumbency without resistance to the plantar surface (no muscle discharges) and with resistance to the plantar surface (rhythmic muscle discharges at a frequency of 6 Hz). The EMG results confirmed the nature of the observations to be consistent with the definition of a tremor.

**VIDEO 2:** Video of the electromyogram of the quadriceps femoris muscle of the conscious cat in standing position.

The clinical and EMG findings were interpreted to be consistent with a diagnosis of a postural tremor, specifically pseudo‐OT. Differentiation between primary pseudo‐OT and pseudo‐OT plus was not possible, because underlying causes including degenerative central nervous system (CNS) disorders, inflammatory diseases, and unidentified toxic or metabolic disorders could not be excluded.

Initially, the cat was left untreated because the tremor did not seem to have any effect on the functional use of the limbs or quality of life. However, because the tremors in the pelvic limbs seemed to increase in severity (amplitude) over the next 3 months, a trial treatment was started using gabapentin 10 mg/kg PO q8h for 2 weeks and 20 mg/kg PO q8h for 1 week. Treatment had no effect on the OT over the course of 3 weeks. Sedation was noted as an adverse effect. Treatment with gabapentin was discontinued and clonazepam 0.1 mg/kg PO q12h was prescribed. After administration of clonazepam, the tremor did not improve and the cat showed marked signs of ataxia and polyphagia, which were considered to be adverse effects of the clonazepam. Therefore, this treatment was discontinued after 1 day and long‐term effectiveness could not be evaluated.

Clinical follow‐up (2), videos (Video 1), and phone calls during the next 12 months disclosed variable tremor amplitude (possibly corresponding to difference in outdoor temperatures [worse when cold, better when warm]) and difficulty rising, sitting, and jumping as well as a wide‐based stance to a variable degree in association with tremor development. Overall, these features waxed and waned but did not indicate a clearly progressive underlying disorder. The cat continued to be ambulatory, playful, and otherwise neurologically normal.

## 3 DISCUSSION

Recently, a study retrospectively evaluating different types of tremors in cats was published.^13^ The most common diseases in cats with tremors were degenerative encephalopathies, hepatic encephalopathy caused by congenital portosystemic shunts, feline infectious peritonitis, or intoxication. Postural tremors of the pelvic limbs were not included in that study and have not been described in cats.

In the case we report here, the cat was confirmed to have tremors based on the results of EMG in the conscious state in standing position, showing rhythmic muscle discharges. The tremor was action‐related (not present at rest), postural, and most obvious when standing (orthostatic), but also present during the weight‐bearing phase of ambulation and when pressure was placed on the plantar surface of the paw to mimic opposition of extensors to gravity. The EMG‐determined frequency of 6 Hz was consistent with a diagnosis of pseudo‐OT.

Orthostatic tremors can be subdivided into primary OT and primary OT plus and have a tremor frequency of 13 to 18 Hz. This particular frequency is included in definitions in much of human as well as veterinary medical literature.^1^, ^2^, ^3^, ^4^, ^5^ The cat described here showed typical signs of OT, but EMG showed a low tremor frequency of 6 Hz. Interestingly, pseudo‐OT in humans often is characterized by a 6 to 7 Hz frequency.^4^ In the human medical literature, this phenomenon of low‐frequency (3‐12 Hz) tremors has been referred to with different terminology including pseudo‐OT, slow OT, tremor in orthostatism, and low‐frequency OT.^1^, ^4^, ^12^ The term pseudo‐OT is preferred in a recent consensus report.^1^ The term pseudo‐OT may prevent physicians from actually diagnosing slow OT (ie, the term “pseudo” suggests that it looks like, but actually is not, an OT).^12^ From our perspective, taking into account the human medical literature,^1^, ^12^, ^14^, ^15^ the term slow OT or low‐frequency OT is preferable. However, because relatively little is known about postural tremors in veterinary neurology, we elected to use the term pseudo‐OT in this report based on the most recent consensus report in the human medical literature.^1^

In dogs, only 13 to 18 Hz OT has been reported. In 1 study, 88% of dogs were not diagnosed with an underlying disorder.^5^ It mostly occurs at a young age, with large breed dogs overrepresented.^5^, ^6^, ^8^, ^11^ A case series showed that 80% of dogs were <2 years old when clinical signs started, with a median age of 12 months.^5^ More than half of owners described worsening of the condition over time. In our case, the progression of signs initially was reported, but during the follow‐up period waxing and waning were noticed.

The most frequently presenting clinical signs of OT in dogs are comparable to what was found in our case: limb tremors while standing and difficulty rising or sitting (noted during follow‐up). In the majority of cases in dogs, all 4 limbs were affected.^5^, ^6^, ^9^ This finding contrasts with our case, where the cat only showed pelvic limb tremors.

A few features in our cat deserve discussion. The first is the fact that the tremors were noticeable in the pelvic limb that supported weight when the cat was walking. This finding might be a result of the subjectively large amplitude coupled with low frequency, enabling appreciation of this feature (Video 1). Visually, the limbs did not exhibit a tremor during the protraction phase but we cannot exclude that it was present. In the veterinary literature, OT is reported to disappear when walking.^5^ However, OT research in humans has shown that tremors persist when the patient is walking.^16^ Tremors tend to increase in frequency and usually are not observed by eye during walking, but are confirmed on ambulatory EMG.^16^ The relatively low frequency and high amplitude of the tremors in our case might have resulted in its being conspicuous when the cat was walking and using the supporting limb. We could not perform ambulatory EMG in our cat. Second, no “dancing sign” was noticed in our cat.^5^ This feature was reported in dogs with high‐frequency OT. Whether or not this sign would be lacking in pseudo‐OT in dogs as well as in our cat remains to be determined. Finally, difficulties jumping were reported in our cat. Postural instability in humans with OT has been reported and it has been shown that people with tremors of <10 Hz have more difficulty walking and fall more frequently compared with patients who have tremor frequencies >10 Hz.^4^ Differences in bipedal posture and ambulation versus quadrupedal posture and ambulation might play a role in the observation of such signs in veterinary patients. The tendency to lie down and difficulty jumping reported in our cat may reflect this observation in human patients. The act of jumping would require more fine‐tuned action of muscles, especially those of the pelvic limbs which were affected by OT in our case, and might have added to the difficulty with jumping as noticed.

Regarding humans, typical signs of OT include the tremor itself, hem sign (fast trembling of the clothing hem covering the thigh), an abnormal tandem gait, walking with bent knees, and wide‐based walking.^17^ A predisposition in females has been reported for OT as well as pseudo‐OT.^12^, ^18^ A retrospective study in humans showed that, in 79% of people with primary OT, signs progressed over time with some patients even developing neurological signs, whereas tremor frequency did not change.^5^, ^18^ One‐third to approximately two‐thirds of human patients with pseudo‐OT do not show any other neurological signs.^4^, ^12^

Currently, no consensus exists for the treatment of primary OT in veterinary medicine. In dogs, treatment of primary OT using gabapentin, pregabalin, phenobarbital, primidone, or clonazepam can result in a decrease or remission of tremors in 85% of cases.^5^ However, in 15% of dogs, tremors worsened. No significant difference has yet been found for the different types of medications used.^5^ In humans, treatment of primary pseudo‐OT with clonazepam, gabapentin, or primidone is mostly used and can lead to partial remission of tremors. Overall, efficacy rates varied between 51% and 100%.^12^, ^17^ Clonazepam and gabapentin were effective among all tremor frequencies, whereas primidone was only effective in frequencies of 5 to 10 Hz.^12^ Other treatments include levodopa, carbidopa, pregabalin, propranolol, and topiramate.^17^ Importantly, both in humans and dogs, treatment can be disappointing. Results are inconsistent because not all patients respond to treatment, and adverse effects can occur.^4^, ^5^, ^6^, ^9^, ^17^ Our cat was first treated with gabapentin at a dosage of 10 mg/kg PO q8h. However, this treatment did not lead to any improvement, and the dose was increased but discontinued after 1 more week without effect. Sedation was noticed as an adverse effect. Therefore, a trial with clonazepam 0.1 mg/kg PO q12h was started. This treatment also did not seem to improve the clinical condition and resulted in notable adverse effects (severe ataxia and increased appetite), leading to discontinuation of the treatment after just 1 day.

Our report has some limitations. These include the lack of additional diagnostic tests, such as advanced imaging (magnetic resonance imaging) of the CNS or cerebrospinal fluid analysis, lack of long‐term evaluation of the treatments that were employed, and lack of histopathological examination of the CNS. These limitations preclude a classification as primary pseudo‐OT or primary pseudo‐OT plus, because we cannot exclude the possibility of an underlying neurodegenerative disorder. Many of these same limitations apply to the veterinary reports on OT in dogs.^5^, ^6^, ^7^, ^8^, ^9^, ^10^, ^11^ Nonetheless, the reports on OT in dogs and our report on pseudo‐OT in a cat advance our knowledge of this type of postural tremor in these species.

In conclusion, we describe the first case of pseudo‐OT in a cat. It shows that, as in humans, OT can occur at a frequency <13 Hz and that it is a possible differential diagnosis in cats with tremors. Because it is a single case, no definitive conclusions can be drawn. More research is necessary to fully understand this type of OT in veterinary medicine.

## CONFLICT OF INTEREST DECLARATION

Authors declare no conflict of interest.

## OFF‐LABEL ANTIMICROBIAL DECLARATION

Authors declare no off‐label use of antimicrobials.

## INSTITUTIONAL ANIMAL CARE AND USE COMMITTEE (IACUC) OR OTHER APPROVAL DECLARATION

Authors declare no IACUC or other approval was needed.

## HUMAN ETHICS APPROVAL DECLARATION

Authors declare human ethics approval was not needed for this study.
